# Precise and efficient silencing of mutant Kras^G12D^ by CRISPR-CasRx controls pancreatic cancer progression

**DOI:** 10.7150/thno.46642

**Published:** 2020-09-16

**Authors:** Wang Jiang, Hao Li, Xiyu Liu, Jianping Zhang, Wuhu zhang, Tianjiao Li, Liang Liu, Xianjun Yu

**Affiliations:** 1 Department of Pancreatic Surgery, Fudan University Shanghai Cancer Center, Shanghai 200032, PR China; 2 Department of Oncology, Shanghai Medical College, Fudan University, Shanghai 200032, PR China; 3 Shanghai Pancreatic Cancer Institute, Shanghai 200032, PR China; 4 Pancreatic Cancer Institute, Fudan University, Shanghai 200032, PR China; 5 Department of Breast Surgery, Fudan University Shanghai Cancer Center, Shanghai 200032, PR China; 6 Department of Nuclear Medicine, Fudan University Shanghai Cancer Centre, Shanghai 200032, PR China

**Keywords:** PDAC, Kras^G12D^, CasRx, off-target, gRNA.

## Abstract

**Rationale:** Pancreatic ductal adenocarcinoma (PDAC) is a highly lethal disease with few therapeutic targets and rare effective treatments. Over 90% of PDAC tumors bear a Kras mutation, and the single-site mutation G12D (Kras^G12D^) is most prevalent.

**Methods:** Here, we applied the CRISPR-CasRx system to silence the mutant Kras^G12D^ transcript in PDAC cells. We also used a capsid-optimized adenovirus-associated virus 8 vector (AAV8) to deliver the CRISPR-CasRx system into PDAC orthotopic tumors and patient-derived tumor xenografts (PDX).

**Results:** Our data showed that guided by a KrasG12D-specific gRNA, CasRx is able to precisely and efficiently silence the mutant KrasG12D expression in PDAC cells. The knockdown of mutant KrasG12D by CasRx abolishes the aberrant activation of downstream signaling induced by mutant KrasG12D and subsequently suppresses the tumor growth and improves the sensitivity of gemcitabine in PDAC. Additionally, delivering CasRx-gRNA via AAV8 into the orthotopic KrasG12D PDAC tumors substantially improves the survival of mice without obvious toxicity. Furthermore, targeting KrasG12D through CasRx suppresses the growth of PDAC PDXs. In conclusion, our study provides a proof-of-concept that CRISPR-CasRx can be utilized to target and silence mutant KrasG12D transcripts and therefore inhibit PDAC malignancy.

## Introduction

Genetic alterations (e.g., mutations, amplifications, rearrangements, etc.) often result in constitutive activation of oncogenes, which drive cancer progression. Among the list of oncogenes, *KRAS,* which encodes a small GTPase called Kras 1, might be the most prevalent oncogene in human cancers. *KRAS* mutation occurs in approximately 90% of pancreatic cancers, 30% to 40% of colon cancers, and 15% to 20% of lung cancers, as well as in other cancer types 2. *KRAS* mutation aberrantly activates its downstream signaling pathways, thus contributing to the promotion and maintenance of cancer malignancy 3. Unlike successful anti-tumor targeted inhibitors, e.g. Gefitinib that targets EGFR 4, the development of clinically-approved drugs against mutant Kras has been frustrating. Although recent advances in developing Kras^G12C^-specific inhibitors have brought hope 5, there are no available effective inhibitors against Kras^G12D^. Except for lung cancer, Kras^G12D^ mutation occurs more frequently compared to Kras^G12C^ 6.

Kras^G12D^ mutation is most prevalent in pancreatic ductal adenocarcinoma (PDAC), which is a dismal disease with the mortality closely parallel to its incidence 7. In addition, PDAC patients with *KRAS* G12D-mutant tumors have particularly poor outcomes compared to those with other *KRAS* mutants 8. Therefore, developing a novel therapeutic strategy targeting Kras^G12D^ with potency and safety is urgent for improving PDAC patients' survival. Repairing cancer-associated mutations via genome-editing tools, e.g., the CRISPR-Cas9 system 9, or via siRNA/shRNA offers a concrete possibility for the anti-Kras^G12D^ 10-12*.* However, the unexpected off-target effects associated with these above techniques may limit their potential applications 13. CasRx, which is a newly identified Cas enzyme from RNA-guided, RNA-targeting CRISPR systems, exhibits high efficiency and specificity for transcriptome engineering 14. Furthermore, the off-target effects of CasRx on non-target transcripts was proved extremely low in mammalian cells and the size of CasRx is quite smaller than Cas9 14, highlighting its future utility for therapeutic purpose 15. In this study, we evaluated the potency of the CasRx-mediated knockdown of Kras^G12D^ in PDAC. Besides, for evaluating its therapeutic potential, we delivered CasRx and Kras^G12D^-specific gRNA via the capsid optimized adenovirus associated virus 8 (AAV8) vector into the PDAC orthotopic mice and patient-derived tumor xenografts (PDX) model.

## Results

### CasRx specifically silences mutant Kras^G12D^ in PDAC cells

**Figure 1:**
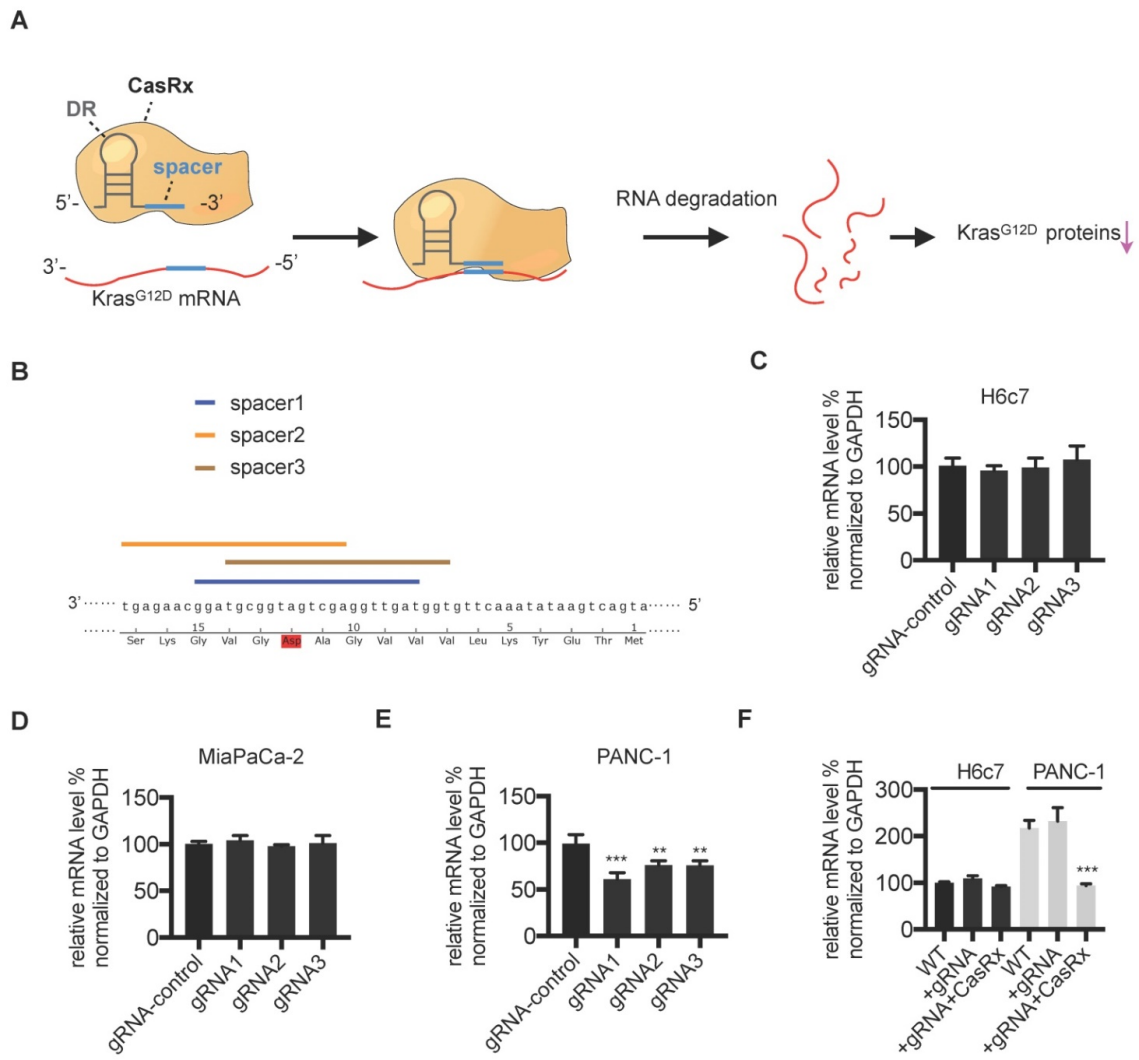
Design and validation of gRNA targeting the mutant Kras^G12D^ transcript. (A) Schematic representation of the mechanism of the CasRx-gRNA system in silencing the mutant Kras^G12D^ transcript. DR, direct repeat. (B) Design of the spacer sequence. Three candidates covering the single mutation G>A in mutant Kras^G12D^ transcript were selected. (C-E) RT-qPCR quantification of Kras mRNA in CasRx-gRNA transiently expressing cells. Plasmids containing either CasRx or gRNA were transiently transfected into cells using lipofectamine3000. gRNA-control represents the gRNA targeting mCherry transcript. One-way ANOVA analysis was used to test the difference between each experimental group and the gRNA-control group. (F) RT-qPCR quantification of Kras mRNA in CasRx-gRNA stably expressing cells. Lentivirus containing either CasRx or gRNA was used to transfect the cells, which were further screened by puromycin selection or flow cytometry sorting. Data represent mean ± SD of three independent assays. One-way ANOVA analysis was used to test the differences between each experimental group and the control group. ^*^: P<0.05; ^**^: P<0.01; ^***^: P<0.001.

We aimed to apply the CasRx system to knock down the mutant Kras^G12D^ transcript in PDAC cells (Figure 1A). First, we verified the potency of the CasRx system in silencing mRNA transcripts by testing its knock-down effect on mCherry expression. As expected, compared to control cells, the transfection of CasRx and mCherry transcript-specific gRNA resulted in a dramatic decrease of mCherry expression in 293T (Figure S1). To specifically target the Kras^G12D^ transcript, three gRNAs with the spacer (22nt) covering the region of single mutated nucleoside A of Kras^G12D^ transcript were selected as candidates (Figure 1B). Human PDAC cells PANC-1 bearing the Kras^G12D^ mutation, MIAPaCa-2 bearing the Kras^G12C^ mutation 16, and the normal human pancreatic ductal epithelial cells H6c7 17 were chosen. The Kras mutation status in these cell lines was confirmed by Sanger sequencing (Figure S1B). Consistent with a previous study 18, the missense mutation of codon 12 in PANC-1 is heterozygous (p.G12D; GGT > GAT), i.e., PANC-1 bears both wild-type (WT) Kras and Kras^G12D^ transcripts (Figure S1B). We transiently transfected PANC-1, MIAPaCa-2, and H6c7 with two plasmids containing CasRx and gRNA. Although the transfection efficiency was not very high (approximately 20%~30% GFP-positivity, data not shown), the cotransfection of CasRx and gRNA candidates resulted in a significant reduction of *KRAS* mRNA in PANC-1 but not in H6c7 or MIAPaCa-2 (Figure 1C-E). gRNA1 presented the best efficiency and hence was chosen for the following experiment (Figure 1E). Note that primers for qPCR cannot discriminate mutant *KRAS* transcripts from the WT transcript. To improve the transfection efficiency, we applied the lentivirus transfection system and obtained the CasRx^+^/gRNA^+^ cells through dual-selection by puromycin and flow-sorting. RT-qPCR analysis showed that the level of total *KRAS* transcripts in CasRx^+^/gRNA^+^ PANC-1 was reduced by more than 50% compared to the control (Figure 1F). In contrast, the level of *KRAS* mRNA in CasRx^+^/gRNA^+^ H6c7 or CasRx^+^/gRNA^+^ MIAPaCa-2 did not significantly decrease (Figure 1F, Figure S3A). These data indicated that CasRx-gRNA is able to specifically silence mutant Kras^G12D^ transcripts in PDAC cells.

### CasRx-gRNA inactivates Kras^G12D^-induced aberrant downstream signaling

**Figure 2:**
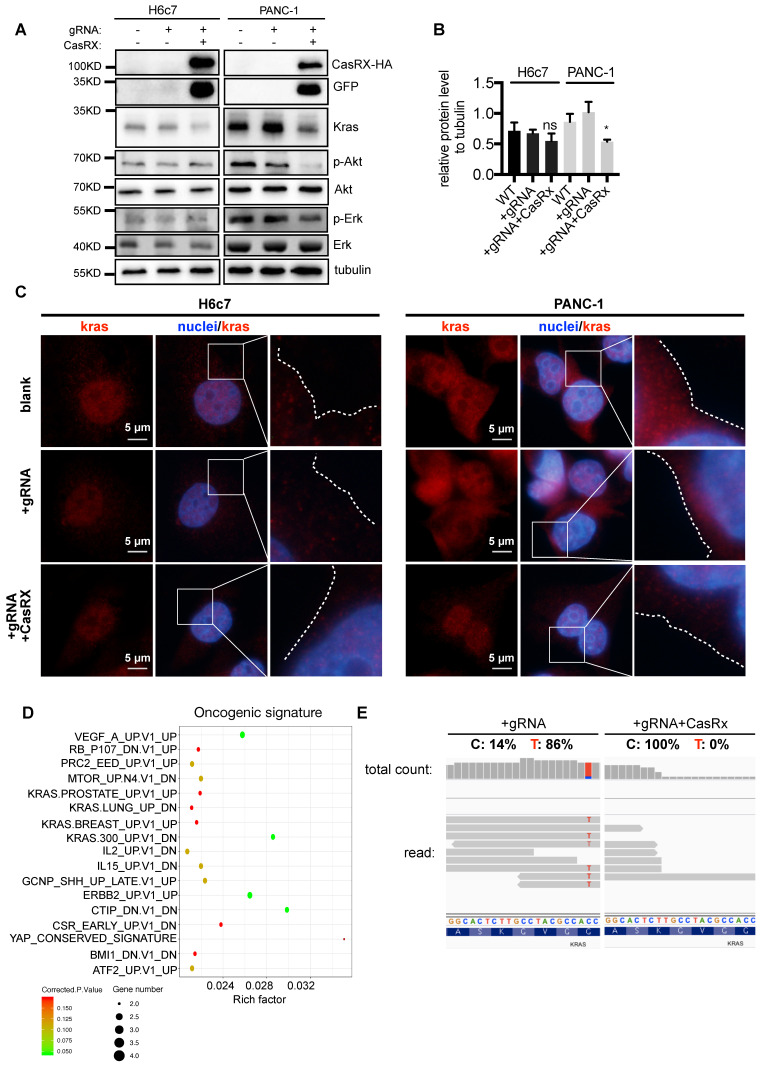
CasRx Stably silences mutant Kras^G12D^ and results in the blockade of the Kras^G12D^ mutation aberrantly activated signaling. (A) CasRx-gRNA stably silences the expression of mutant Kras^G12D^. Indicated cells were analyzed by western blotting. (B) Quantification of the Kras protein level. The band intensities of Kras and tubulin were detected by three-independent Western-blot analysis and estimated by Fiji. The intensity of tubulin was used for normalization. The Data represent the means of three independent assays. One-way ANOVA analysis was used to test the differences between each experimental group and control group. (C) CasRx-gRNA inhibits the plasma-membrane location of Kras in PANC-1. Immunofluorescence was used to detect the distribution of Kras by using anti-Kras antibody. White dotted lines indicate the cell contour. Nucleus was stained by DAPI. (D-E) Transcriptomic alterations analysis by RNAseq. CasRx^-^/gRNA^+^ PANC-1 cells were used as a control for comparison. (D) Pathways of significantly downregulated genes from CasRx^+^/gRNA^+^ PANC-1 enriched in oncogenic signature compared to CasRx^-^/gRNA^+^. Rich factor: the ratio of differentially expressed gene numbers annotated in this pathway to all gene numbers of this pathway. Corrected.P.Value: P value corrected by Benjamini and Hochberg. Gene number: downregulated gene numbers annotated in this pathway. (E) Representation of reads sequence containing the region of G12D mutation in Kras transcript. Two replicates were used for RNA-seq.^ *^: P<0.05; ^**^: P<0.01; ^***^: P<0.001.

Consistent with the above data, CasRx-gRNA resulted in a dramatic reduction of Kras protein in PANC-1 cells (Figure 2A-B) but not in H6c7 or MIAPaCa-2 cells (Figure 2A-B, Figure S3B). To recruit and activate downstream signaling pathways, Kras must localize primarily to the inner leaflet of the plasma membrane (PM) 19. Compared to the control, we observed an obvious reduction of accumulated Kras protein near PM in CasRx^+^/gRNA^+^ PANC-1 (Figure 2C, Figure S2A-B). Additionally, the protein levels of p-Akt and p-Erk, which are downstream factors upregulated by Kras activation, were much lower in CasRx^+^/gRNA^+^ PANC-1 (Figure 2A). Note that CasRx overexpression alone, without gRNA, did not affect the Kras expression (Figure S2C). We further performed RNAseq to determine the transcriptomic alteraction. CasRx targeting mutant *KRAS*^G12D^ transcripts significantly down-regulated the expression of *KRAS* (fold change ~ 4) and *KRAS* signaling-related genes, e.g. SERPINA3, IGF2, and PCSK1N (Figure S2D). Besides, down-regulated genes in PANC-1 were enriched mainly in the *KRAS* signaling, *ERBB2* signaling, and mTOR signaling from the oncogenic signature (Figure 2D). In H6c7 (*KRAS* WT cell), RNA-seq data showed that only a limited number of genes were altered, suggesting a low off-target effect of CasRx system in Kras WT cells (Figure S2E). Intriguingly, we found that reads of mutant Kras^G12D^ transcript could not be detected in CasRx^+^/gRNA^+^ PANC-1 by RNA-seq while the proportion of mutant Kras^G12D^ transcript was approximately 86% in CasRx^-^/gRNA^+^ PANC-1 (Figure 2E). To further confirm the efficiency of the CasRx-gRNA system in silencing the mutant Kras^G12D^ transcript, we targeted the Kras transcripts in AsPC-1 cell, which owns a homogenous Kras^G12D^ mutation (Figure S1B). The data showed that the knockdown efficiency of *KRAS* transcripts by CasRx-gRNA in AsPC-1 was approximately 90% (Figure S4A). Furthermore, the CasRx system reduced the protein levels of Kras, p-Erk and p-Akt in AsPC-1 cells (Figure S4B). Together, these data suggested that CasRx is able to specifically and potently silence Kras^G12D^ mRNA transcript and therefore inactivate the Kras^G12D^ downstream signaling.

### Silencing mutant Kras^G12D^ inhibits PDAC cells proliferation and improves the sensitivity to Gemcitabine

**Figure 3:**
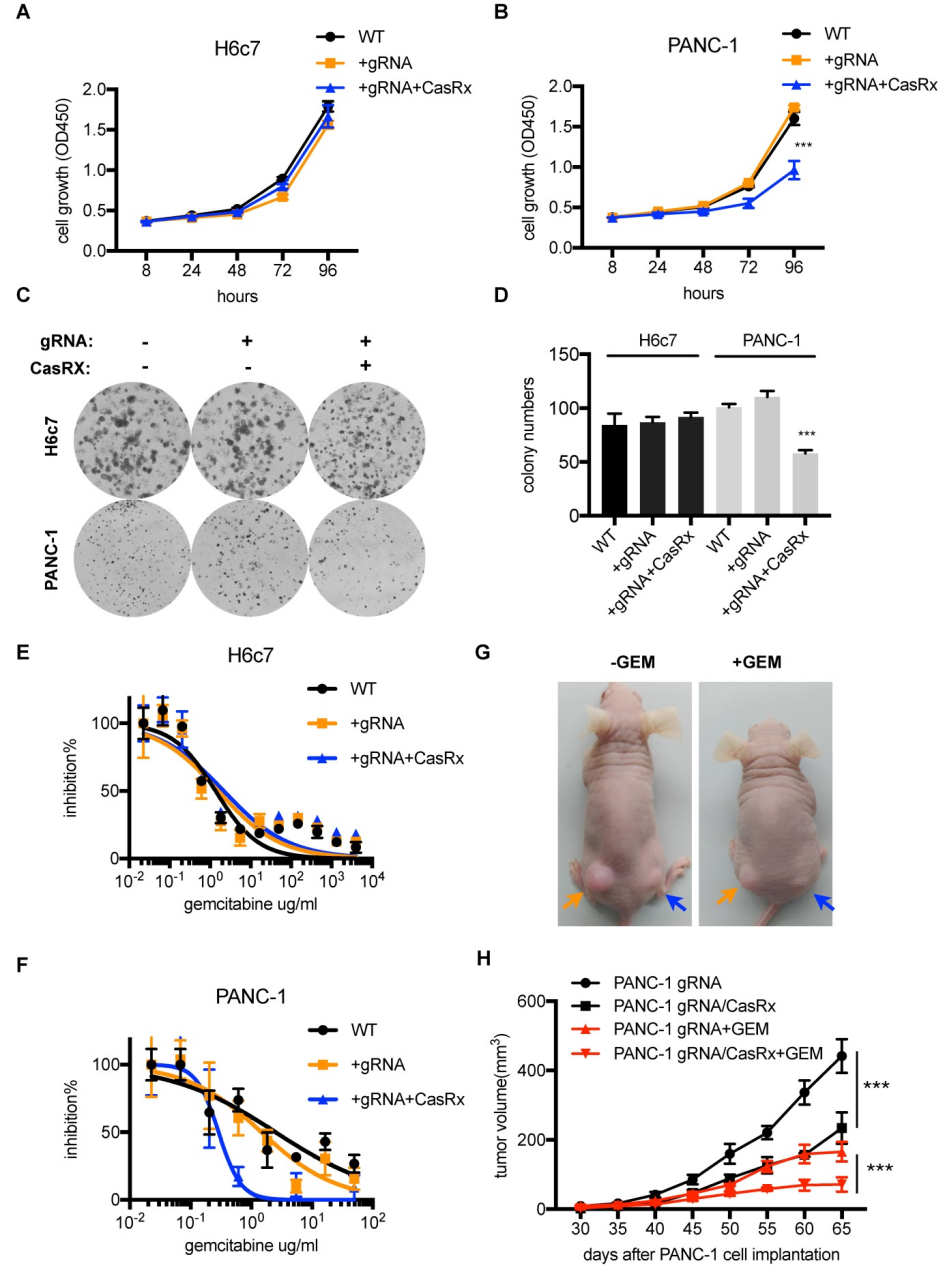
The knockdown of mutant Kras^G12D^ inhibits pancreatic ductal adenocarcinoma (PDAC) cell proliferation and improves the GEM sensitivity. (A-B) Cell proliferation analysis of H6c7 and PANC-1 cells. CCK-8 was used to quantify the cell viability. Two-way ANOVA analysis was used to compare the main curve effects. (C) CasRx-gRNA inhibits colony-formation of PANC-1 but not H6c7. (D) Quantification of colony formation. Data represent the means of three independent assays. One-way ANOVA analysis was used to test the difference between each experimental group and the control group. (E-F) Determination of the cell sensitivity to gemcitabine. Serial diluted GEM was added, and the cell viability was detected by CCK-8 analysis. (G) Representation of tumors in nude mice (n=5). gRNA^+^ PANC-1 (orange arrow) and CasRx^+^/gRNA^+^ PANC-1(blue arrow) were subcutaneously injected to the left and right flanks of the nude mice. Note that the initial tumor growth of PANC-1 was slow in the first month. (H) Statistics of tumor growth with/without GEM treatment. GEM was added intraperitoneally one month after the cell injection. The effect of PANC-1 gRNA/CasRx versus PANC-1 gRNA, and the effect of PANC-1 gRNA/CasRx+GEM versus PANC-1 gRNA+GEM were tested by two-way ANOVA to compare the main curve effects.^ *^: P<0.05; ^**^: P<0.01; ^***^: P<0.001.

Several studies have demonstrated that targeting mutant Kras was able to suppress PDAC cell proliferation and improve the sensitivity to chemotherapeutic drugs 11, 20. Next, we tested the anti-tumor effect of CasRx-gRNA in PDAC. We found that the stable expression of CasRx and gRNA resulted in approximately 50% inhibition of cell proliferation of PANC-1, while it did not affect the proliferation of H6c7 and MIAPaCa-2 cells (Figure 3A-D, Figure S3C-D). In addition, we found that CasRx-gRNA reduced the half-maximal inhibitory concentration (IC50) of Gemcitabine (GEM) in PANC-1 by approximately 10 fold, while did not alter the sensitivity of GEM in H6c7 and MiaPaCa2 (Figure 3E-F, Figure S3E). Consistent with this result, the CasRx system inhibited cell proliferation and improved the sensitivity of GEM in AsPC-1 cells (Figure S4C-E). To test the effect of CasRx on PDAC tumor growth, we subcutaneously injected the CasRx^-^/gRNA^+^ PANC-1 and CasRx^+^/gRNA^+^ PANC-1 cells to the left and right flanks of BALB/c nude mice, respectively (Figure 3G). Of note, the initial tumor growth of the PANC-1 xenograft was slow and one month later the tumor became measurable. The data showed that the knockdown of mutant Kras^G12D^ by CasRx-gRNA significantly suppressed the PANC-1 tumor growth (Figure 3G-H) and sensitized tumors to GEM (Figure 3G-H, Figure S5A-C). Compared to the *in vivo* phenotype, the CasRx/gRNA-induced growth inhibition *in vitro* seemed limited. We speculated that this may have resulted from the anchorage dependence of sustained *KRAS* expression in cancer cells 21. To confirm this, we detected the PANC-1 cell growth under a 3D spheroid culture. The stable knockdown of mutant Kras^G12D^ in PANC-1 by CasRx-gRNA substantially reduced the spheroid growth (Figure S5D). Together, these data indicated that the CasRx-gRNA-based knockdown of mutant Kras^G12D^ is able to suppress tumor growth and improve the sensitivity to GEM in PDAC tumors.

### AAV8-delivery of CasRx-gRNA prolongs the survival of mice orthotopically implanted with Kras^G12D^ PDAC tumors

**Figure 4:**
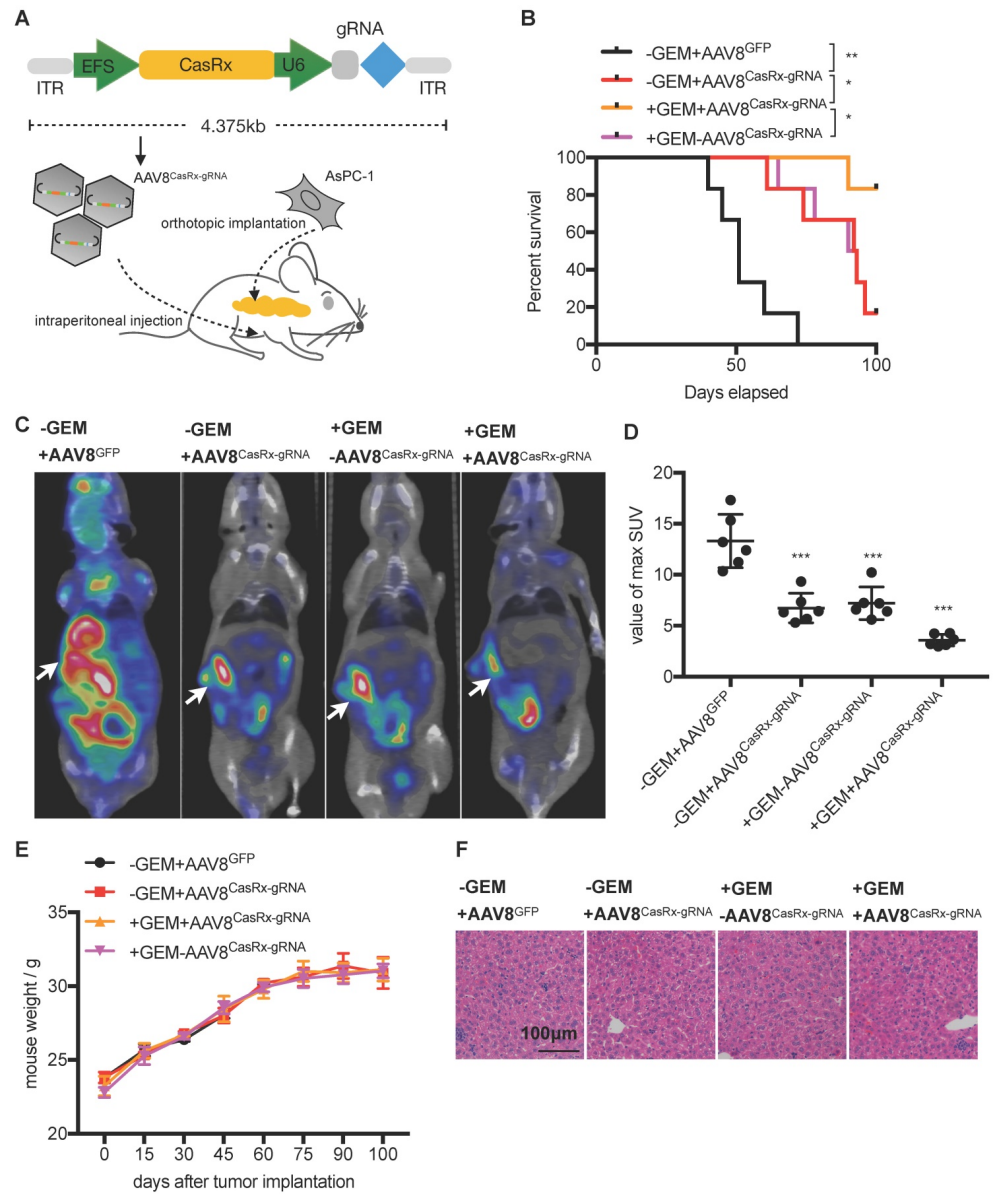
The AAV8-mediated delivery of CasRx-gRNA controls pancreatic ductal adenocarcinoma (PDAC) tumor malignancy in the orthotopic mouse model. (A) Schematic representation of the AAV8-mediated delivery of CasRx-gRNA system. AsPC-1 cells bearing a homogenous Kras^G12D^ mutation were orthotopically injected into the pancreas of nude mice BALB/c (n=5). (B) CasRx-gRNA improved the mouse survival. The log-rank test was used for survival curve comparison. (C) Representative PET-CT images of the orthotopic AsPC-1 tumors. (D) Statistics of maximum SUV of orthotopic AsPC-1 tumors as scanned by PET-CT. (E) Weight of the orthotopic mouse. (F) H&E staining of liver tissue. One-way ANOVA analysis was used to test the differences between each experimental group. ^*^: P<0.05; ^**^: P<0.01; ^***^: P<0.001.

The CasRx protein consists of 996 amino-acids, making it quite smaller than the Cas9 protein and is more suitable to be delivered by the adeno-associated virus (AAV) vector 22. We chose a capsid-optimized Y447+Y733F AAV8 vector 23 to specifically deliver CasRx-gRNA into the pancreas (Figure 4A). The human PDAC cell-line AsPC-1 was orthotopically implanted into the mouse pancreas, and 15 days later, AAV8^CasRx-gRNA^ was intraperitoneally injected (AAV8^GFP^ was set as the negative control) (Figure 4A). One week later, treatment with GEM was initiated each week via intraperitoneal injection. Compared to the control, the 100-day survival rate of mice treated with AAV8^CasRx-gRNA^ increased by up to 33%, and GEM treatment further prolonged the mice's survival by 2-fold (Figure 4B). Of note, AAV8^CasRx-gRNA^ was able to reduce 50% of the Kras mRNA in the orthotopic tumor (Figure S4F). Besides, about one month later, we imaged the orthotopic PDAC tumor by positron emission computed tomography combined with computed tomography (PET-CT). The maximum standard uptake value (SUV) of orthotopic tumors from mice treated with AAV8^CasRx-gRNA^ was significantly lower than that of the control group (Figure 4C-D), suggesting that AAV8^CasRx-gRNA^ alleviated the tumor metabolic activity in mice. Of note, AAV8^ CasRx-gRNA^ delivery did not significantly affect the mice's weight or induce observable damage to the liver (Figure 4E-F). Together, these data suggested that the AAV-delivery of CasRx-gRNA is able to control the malignancy of PDAC tumors with relative safety.

### AAV8^CasRx-gRNA^ inhibits the progression of patient-derived xenografts bearing mutant Kras^G12D^

**Figure 5:**
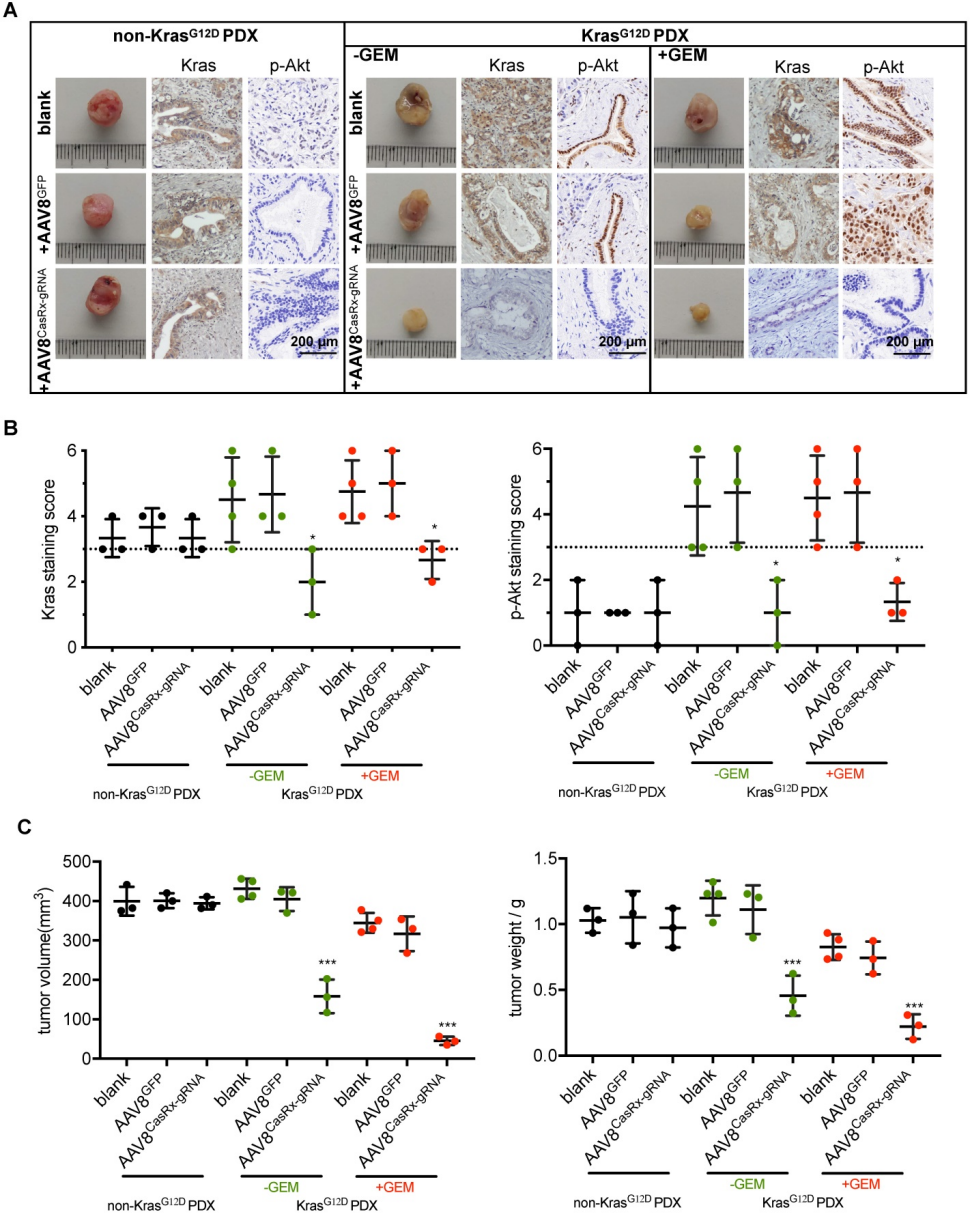
The AAV8-mediated delivery of CasRx-gRNA inhibits the tumor growth of patient-derived tumor xenografts (PDXs). The PDX model was established as described in methods. PDXs bearing non-Kras^G12D^ mutation were used as negative controls. (A) Representative image of PDXs. IHC was used to detect the protein levels of Kras and p-Akt. PDXs bearing no Kras^G12D^ mutation and PDXs bearing Kras^G12D^ mutations were shown. (B) Kras (left) and p-Akt (right) expression scores. IHC staining was described in methods. (C) The volume of PDXs (left) and the weight of PDXs (right) with the indicated treatments. B-C: One-way ANOVA analysis was used to test the difference between each experimental group and its blank group.^ *^: P<0.05; ^**^: P<0.01; ^***^: P<0.001.

To test the clinical application of the CasRx-gRNA system in PDAC patients, we determined the effect of AAV8^CasRx-gRNA^ on PDXs. The non-Kras^G12D^-mutated PDXs were set as a negative control. The AAV8 virus was directly injected into the PDXs as described in methods. As expected, we found that AAV8^CasRx-gRNA^ efficiently and specifically downregulated the expression of Kras, p-Akt and p-Erk in PDXs bearing the *KRAS* G12D mutation but not in PDXs without the *KRAS* G12D mutation (Figure 5A-B, Figure S6). Compared to the blank or AAV8^GFP^-treated PDXs, AAV8^CasRx-gRNA^ either alone or combined with the GEM substantially inhibited the growth of PDXs (Figure 5C). Therefore, these data highlighted the potential application of the CasRx system in targeting the mutant Kras^G12D^ transcript in PDAC patients bearing the *KRAS* G12D mutation.

## Discussion

Gene editing methods that repair cancer aberrant mutation are promising for controlling tumor malignancy and improving patients' survival 24. In this study, we tested the possibility of applying the novel-developed CRISPR-CasRx system to control PDAC progression. A previous study suggested that CasRx exerted 80%~90% knockdown efficiency of the WT *KRAS* transcript in 293T cells 14. Consistent with this, the knockdown efficiency of the mutant Kras^G12D^ transcript in PANC-1 was more than 86% as shown by RNA-seq and over 90% in AsPC-1. Therefore, although a small amount of WT Kras protein remains in PANC-1 cells, CasRx is able to block the Kras^G12D^-induced aberrant activation of signaling pathways involved in PDAC malignancy.

Targeting Kras-associated pathway e.g. blocking the Kras membrane association, exploiting *KRAS*-related metabolism, or by simultaneously inhibiting SHP2 and the Kras downstream factor MEK, may be able to induce the regression of mutant *KRAS* tumor 25. Disappointingly, many of these treatments are under preclinical studies or have ceased development at the phase I/II stage. For example, in contrast to the Kras^G12C^ inhibitor ARS-1620 that elicits submicromolar potency 26, a newly discovered Kras^G12D^ inhibitor that directly binds to a shallow pocket on the surface of Kras^G12D^ inhibits the proliferation of Kras mutant cells with an EC50 of approximately 6.7 μM 27. In this study, we used the CasRx-gRNA system to silence mutant Kras at the mRNA level. We found that the CasRx system exhibits a potent anti-tumor effect on PDAC tumors by specifically targeting the mutant Kras^G12D^ transcript. Future clinical trials to determine its safety and efficacy are warranted.

Compared to the siRNA-mediated knockdown or CRISPR-Cas9-based knockout strategy 28, 29, this CRISPR-CasRx system owns several advantages. First, unlike CRISPR-Cas9 which directly alters the sequence in genomic DNA, CasRx is an RNA-level editing protein that specifically binds and cleaves target RNA transcripts 14. Second, the off-target effects of CasRx are extremely low in mammalian cells. Third, the protein size of CasRx is quite smaller, which is suitable for the AAV-mediated delivery. Notably, by using the capsid optimized AAV8 that was previously reported to target the pancreas with a higher specificity than that of its progenitor AAV8 23, the CasRx system exhibited high efficacy in silencing the mutant Kras^G12D^ transcript in the orthotopic mouse model and PDX model. It would be interesting to investigate whether this CasRx technique could also be applied to other tumor types with the Kras^G12D^ mutation.

To evaluate the therapeutic potential of AAV8^ CasRx-gRNA^ in PDAC, we constructed the orthotopic pancreatic cancer model in nude mice and PDX models. However, whether the immune response could enhance or attenuate the effect of CasRx is elusive. Of note, we had planned to use the murine pancreatic cancer cell Panc-02 in the immune-competent C57BL/6 mouse as Khvalevsky et al. used these cells as the Kras^G12D^ model cells 10. In contrast to their findings, our sequencing analysis indicated that Panc-02 did not carry the G12D mutation (data not shown). Supporting our data, Wang et al. also showed the absence of mutations in genes such as *KRAS*, *TP53*, and *CDKN2A* in Panc-02 30. Future studies may be needed to test the potency and safety of CasRx-based anti-Kras^G12D^ using other murine G12D Kras pancreatic cancer cell lines or in an immunocompetent mice such as the KPC mice 31, 32.

Last, it would be necessary to address that silencing Kras^G12D^ by AAV-mediated delivery of the CasRx system may cause the development of resistance in PDAC tumors bearing the *KRAS* G12SD mutation. For example, cancer cells not infected by AAV in which Kras^G12D^ is not silenced may have a growth advantage. Besides, it is still very difficult to predict whether the 50% *KRAS* knockdown efficiency by AAV delivery observed in our study could translate to PDAC patients. Given that PDAC tumors have different *KRAS* addictions/dependencies due to their high heterogeneity, the therapeutic efficacy of targeting mutant Kras by using CasRx or siRNA or a compound remains unclear. Therefore, future studies of applying the CasRx system to treat PDAC tumors should consider these issues.

In summary, we developed a novel CRISPR-CasRx system that was able to silence the mutant Kras^G12D^ transcript in PDAC. Our study provided a proof-of-concept that CRISPR-CasRx can be utilized to target driver mutations of cancers *in vitro* and *in vivo*.

## Methods

### Plasmids and antibodies

The lentiviral plasmid containing CasRx (NLS-RfxCas13d-NLS) with 2A-EGFP and the plasmid pUC19 containing the direct repeat sequence of gRNA was obtained from Addgene. The spacer sequence targeting mutant Kras^G12D^ or mCherry RNA transcript was inserted between the BbsI restriction site in pUC19. The spacer of mCherry was CGCCGCCGTCCTCGAAGTTCAT. For lentivirus packaging, gRNA targeting the mutant Kras^G12D^ including a direct repeat and Spacer1 was further inserted into the hU6-driven lentiviral vector pLKO.1 with the puromycin resistance gene. Regarding AAV packaging, the sequences of NLS-CasRx-NLS-HA and gRNA targeting Kras^G12D^, which are driven by EF-1α and hU6 promoter, respectively, or GFP that is driven by EF-1α, were inserted into the AAV2 backbone. The AAV2 backbone vector, pAAV8-rep/cap-Y447+Y733F mutant, and pHelper were obtained from Genewize (Suzhou, China). Antibodies for western-blot analysis: Mouse anti-HA (cat#66006-2-Ig), rabbit anti-Kras (cat#12063-1-AP), mouse anti-tubulin (cat#66031-1-lg), rabbit anti-p-Akt (cat#66444-1-lg), rabbit anti-Akt (10176-2-AP), mouse anti-GFP (cat#66002-1lg), anti-Erk (cat# 11257-1-AP) were purchased from Proteintech; Rabbit anti-p-Erk (cat# 9101S) was purchased from Cell Signaling Technology. Antibodies for IHC: rabbit anti-Kras (cat#12063-1-AP), anti-p-Akt (cat#66444-1-lg), were purchased from Proteintech; Rabbit anti-p-Erk (cat#ab214362) was purchased from abcam.

### Cell culturing

HEK293T, MIAPaCa-2, PANC-1, and H6c7 cells were cultured in DMEM medium supplemented with 2 mM L-glutamine, nonessential amino acids, 100 U penicillin per mL, 100 μg streptomycin per mL and 10% FBS (complete DMEM). AsPC-1 was cultured in RPMI-1640 medium supplemented with 15% FBS. All cells were incubated in a 37 °C incubator that provided 5% CO2. Cells were passaged after the confluence reached 90%.

### Cell transfection

Cells were seeded into 24-well plates overnight for transient transfection of plasmids by using lipofectamine3000 (Invitrogen) according to the manufacturer's protocol. Briefly, for each 24-well plate, 0.75 μL lipofectamine3000 and 1 μL P3000 were mixed with 500ng plasmid suspended in 50 μL Opti-MEM at room temperature for 15 min. The mixture was added to the cells for about 6 h, and later the cell culture was replaced with fresh complete DMEM. About 48 h later, cells were harvested for RT-qPCR or visualized by a fluorescence microscopy.

### RT-qPCR

Cells were washed with 1×PBS once prior to harvesting. The total cellular RNA was extracted using TRNzol Universal Reagent (Tiangen) according to the manufacturer's protocol, and the concentration of the RNA was determined using a NanoDrop 2000. The extracted RNA was reverse-transcribed into cDNA using ReverTra Ace qPCR RT Kit (Toyobo), and cDNA was subjected to qPCR using ABI Q6 using SYBR Green (Toyobo). GAPDH was used as the internal control to create the relative mRNA levels of interested genes of interest by the ΔΔCt method. The Primers (5'-3') used for qPCR were as follows: *GAPDH*: F-CGTGGAAGGACTCATGACCA; R-GCCATCACGCCACAGTTTC. *KRAS*: F-CCTGCTGAAAATGACTGAATATAAACTTGTGGT; R-ATTTATGGCAAATACACAAAGAAAGCCCT.

### Lentivirus packaging and transfection

For lentivirus packaging, psPAX2 (a packaging plasmid), pMD2.G (a G protein-expressing plasmid), and lentiviral vectors were cotransfected into 293T cells using lipofectamine3000 according to the manufacturer's protocol; Then, 24 and 48 h later, the supernatants containing lentivirus were harvested and filtered through 0.45-μm filters. CasRx^+^/gRNA^+^ cells transfected by lentivirus were selected with puromycin (10 μg/mL), and GFP positive cells were further selected via flow-cytometry sorting.

### AAV packaging

The AAV packaging was conducted as previously described 33. Briefly, recombinant AAV (rAAV) vectors were generated by the transient transfection of 293T cells using three AAV plasmids (pAAV8-rep/cap-Y-F mutant, pAAV2-NLS-CasRx-NLS-HA-gRNA/pAAV2-GFP, and pHelper). Then, 293T cells were transiently transfected at 80% confluence using polyethyleneimine. The Cells were collected 72 h posttransfection, lysed, and treated with 25 units/mL of benzonase nuclease. Subsequently, the recombinant AAV was purified by iodixanol-based gradient density centrifugation, followed by column chromatography. The Recombinant AAV vectors were then concentrated to a final volume of 0.5 mL in PBS using Amicon Ultra 10K centrifugal filters (Millipore). The viral DNA-containing AAV vector titers were quantified by real-time PCR analysis. A 10-fold dilution series of the control plasmid DNA (pAAV2-NLS-CasRx-NLS-HA-gRNA) was used to generate a standard curve to determine the AAV vector genome titer (GC/mL). After viral titration (> 1E+13GC/mL), the AAV was aliquoted and stored at -80 °C until used.

### Western blot analysis

Western blot anlysis was performed as previously described (9). Briefly, samples were collected in Laemmli buffer, and total proteins were separated by SDS polyacrylamide gel electrophoresis and transferred onto a PVDF membrane (Millipore). The membrane was blocked using 5% dried milk and incubated with the indicated primary antibody and secondary antibody. Bands were visualized using Omni ECL reagent (EpiZyme) under GE AI600, and the gray intensity was acquired by using Fiji (NCBI).

### Immunofluorescence

Cells grown on glass coverslips in a 24-well plate were fixed with 4% paraformaldehyde for 15 min, permeabilized with 0.5% Triton X-100, and blocked with 5% calf serum. The cells were then incubated with the primary antibodies rabbit anti-Kras (cat#12063-1-AP) for 1 h at room temperature, washed, and incubated with goat anti-rabbit-cy3 for 1 h at room temperature. After washing, the cells were incubated with DAPI for 1 min. Finally, the glass coverslips were mounted onto glass slides with Fluoromount Aqueous mounting medium (Proteintech). Images were acquired captured by fluorescence microscopy (Olympus).

### Cell proliferation

For half-maximal inhibitory concentration (IC50) determination, cells (3 x 10^3^ per well) were seeded into 96-well plates. After 24 h, the cells were treated with the indicated concentration of drug or DMSO for a further three days, respectively. The cell proliferation was determined by a CCK-8 assay according to the manufacturer's protocol (Dojindo). The IC50 was determined according to a dose vs. response curve by GraphPad Prism 6.0.

### Colony-forming assay

Cells were seeded into 24-well plates at a low density of approximately 100 cells per well. The cells were cultured for 2-3 weeks. Cell colonies were stained by 1% crystal violet and then captured and counted by a plate scanning machine. The colony-forming efficiency was calculated by the following formula: (formed-colonies / seeded cells) × 100%.

### RNA-seq analysis for PANC-1

Oligo(dT)-attached magnetic beads were used to purify the mRNA. The purified mRNA was fragmented into small pieces with a fragment buffer at an appropriate temperature. Then, first-strand cDNA was generated using random hexamer-primed reverse transcription, followed by a second-strand cDNA synthesis. Afterward, A-Tailing Mix and RNA Index Adapters were added with incubation for end repair. The cDNA fragments obtained from the previous step were amplified by PCR, and the products were purified by Ampure XP Beads, and then dissolved in EB solution. The products were validated by the Agilent Technologies 2100 bioanalyzer for quality control. The double-stranded PCR products from the previous step were heated denatured and circularized by the splint oligo sequence to get the final library. The single-strand circle DNA (ssCir DNA) was formatted as the final library. The final library was amplified with phi29 to make a DNA nanoball (DNB) which had more than 300 copies of one molecular. The DNBs were loaded into the patterned nanoarray and single end 50 bases reads were generated on the BGIseq500 platform (BGI-Shenzhen, China).

The sequencing data was filtered with SOAPnuke (v1.5.2) by (1) removing reads containing sequencing adapter; (2) removing reads whose low-quality base ratio (base quality less than or equal to 5) is more than 20%; (3) removing reads whose unknown base ('N' base) ratio was more than 5%, afterward clean reads were obtained and stored in FASTQ format. The clean reads were mapped to the reference genome using HISAT2 (v2.0.4). Bowtie2 (v2.2.5) was applied to align the clean reads to the reference coding gene set, and then the expression level of the genes were calculated by RSEM (v1.2.12). Differential expression analysis was performed by 'edgeR' with a false dicovery rate (FDR) < 0.05 and a |Fold Change| > 2. Volcano plot was plotted utilizing the 'EnhancedVolcano' package from R.To take insight to the change of phenotype, Enriched pathways of differentially expressed genes in CasRx+/gRNA+ PANC-1 were analyzed utilizing the 'clusterProfile' package from R. The oncogene signatures were downloaded from 'C6: oncogenic signatures MsigDB' (https://www.gsea-msigdb.org/gsea/msigdb/collections.jsp#H). Significant levels of pathways (P value < 0.05) were selected for plotting. Rich factor indicated the ratio of differentially expressed gene numbers annotated in this pathway to all gene numbers annotated in this pathway. The greater the Rich factor, the greater the degree of pathway enrichment. P values (ranging from 0 to 1) were corrected by Benjamini and Hochberg. Lower corrected P value indicates greater pathway enrichment.

### mRNA sequencing by Illumina HiSeq for H6c7

Each sample was sequenced by Illumina Hiseq according to the manufacture. In brief, the total RNA was extracted using TRIzol Reagent (Invitrogen)/RNeasy Mini Kit (Qiagen)/other kits, followed by library construction. The poly(A) mRNA isolation was performed using Poly(A) mRNA Magnetic Isolation Module or rRNA removal Kit. Then, the libraries were sequenced with an Illumina HiSeq instrument (Illumina, San Diego, CA, USA) using a 2 x 150 bp paired-end (PE) configuration. The pass filter data in fastq format were processed by Cutadapt (V1.9.1) to yield high quality clean data, then aligned to the reference genome via software Hisat2 (v2.0.1). Gene and isoform expression levels were estimated by HTSeq (v0.6.1). Differential expression analysis used the edgeR package from R with FDR < 0.05 and |Fold Change| > 2.

### Animals

Regarding the subcutaneous xenograft model, 8-week-old female BALB/c nude mice were used. Cells (1 × 10^6^) were harvested and resuspended in 100 μL mixture of PBS and were injected directly into the subcutaneous of the mice. The tumor volumes were calculated by V = L × (W × W) / 2, where L is the length (longest dimension) and W is the width (shortest dimension). The dose of gemcitabine used was 100 mg/kg given via intraperitoneally injection.

Regarding the orthotopic pancreatic cancer model, about 3 × 10^6^ AsPC-1 cells were directly injected into the BALB/c nude mouse pancreas. Briefly, the mice were anesthetized intraperitoneally. After local shaving and disinfection, the abdominal cavity was opened with a 1.5-cm-long longitudinal incision into the left upper quadrant. The tail of the pancreas was identified after the spleen was lifted. Then, 100 microliters of AsPC-1 was injected slowly into the pancreatic parenchyma. To prevent further leakage, the needle was kept in the injection site for 30 s prior to removal. At 15 days after implantation, AAV8 virus was intraperitoneally adminisered at a dose of 3×10^11^ GC/animal. At 30 days after implantation, gemcitabine was injected intraperitoneally with at 100 mg/kg every week.

PDAC PDX mouse models were established by using freshly isolated pancreatectomy samples as previously described 33. Briefly, every PDAC sample was isolated in two parts. One part was subjected to sanger sequencing for Kras mutation. The remaining sample was cut into five equal blocks of approximately 10 mm^3^ for subcutaneous transplantation into the flanks of nonobese diabetic severe combined immune-deficient mice. Next, according to the Kras^G12D^ mutation, we defined the samples as belonging to one of the following two groups: Kras^G12D^ mutation (n = 10) and non-Kras^G12D^ mutation (n = 3). Eventually, 29 PDXs were successfully established. Mice with the same patient samples from Kras^G12D^ groups were randomly assigned to the Kras silencing group or the control group. When the PDX reached a mean volume of 100 mm^3^, AAV8^CasRx-gRNA^ or AAV8^GFP^ (3×10^11^ GC/mL) was administered by direct injection into PDXs (2 sites, 10 μL/site). One month later, the mice were prepared for histopathological examination.

All animals received humane care per the criteria outlined in the “Guide for the Care and Use of Laboratory Animals” issued by the National Institutes of Health (NIH publication 86-23 revised 1985).

### PET-CT (positron emission computed tomography combinated with X-computed tomography)

Animal-PET-CT scans and image analyses were performed 1 h after an injection of radiolabeled tracer (via intraperitoneal injection with 5.55 MBq 18F-FDG in 0.1 mL saline) using an Inveon Animal-PET-CT (Siemens Preclinical Solution, Knoxville, TN). Animals were maintained under 2 % isoflurane anesthesia during scanning period. The mice were placed in the prone position on the bed of the scanner (five-min CT scanning followed by ten-min PET scanning). The animal-PET and animal-CT images were generated separately and then fused using Inveon Research Workplace (Siemens Preclinical Solution, Knoxville, TN). Three-dimensional ordered-subset expectation-maximization (OSEM3D)/maximum algorithm was used for image reconstruction. The region of interest (ROI) was manually drawn covering the whole tumor on the fused images for further analysis. The highest uptake point of the entire tumor was included in ROI and no necrosis area was allowed. After the acquisition, SUVmax was assessed on the Siemens syngo MultiModality WorkPlace (MMWP) system by a single nuclear medicine physician. SUVmax was determined by manually placing a cylindrical ROI over the tumor of interest.

### Immunohistology (IHC)

IHC was performed as previously described 34. Regarding the quantification of Kras and p-Akt expression, the staining intensity and the percentage of stained cells were evaluated. The cells with no staining were scored as 0 points, 1 point represented weak staining intensity, 2 points represented moderate staining intensity, and 3 points represented strong staining intensity. Additionally, the percentage of stained tumor cells was assessed as follows: 0% corresponded to 0 points, less than 25% corresponded to 1 point, 25%-50% corresponded to 2 points, and more than 50% corresponded to 3 points. The final score was equal to the sum of the two types of scores. A staining score ranging from 0 to 3 points represented a low expression level, and a score more than 3 points was considered a high expression level.

### Statistical analyses

The continuous variables in different subgroups were compared using an unpaired t-test and a one-way analysis of variance. All the tests were two-sided, and p < 0.05 was considered statistically significant. The statistical analyses were performed using SPSS 24.0 (SPSS Inc., Chicago, IL) or GraphPad. The categories of *, P<0.05; **, P < 0.001; ***, P < 0.0001 were used to represent the p-values.

### Grant support

This work was supported by grants from the National Science Foundation for Distinguished Young Scholars of China (81625016), the National Natural Science Foundation of China (81871941, 81872366, 81827807, 81802675, 81701630 and 81702341), the Outstanding Academic Leader Program of the “Technological Innovation Action Plan” in Shanghai Science and Technology Commission (18XD1401200), the Scientific Innovation Project of Shanghai Education Committee (2019-01-07-00-07-E00057), the Natural Science Foundation of Shanghai (19ZR1410800), Clinical and Scientific Innovation Project of Shanghai Hospital Development Center (SHDC12018109), and the Young Talented Specialist Training Program of Shanghai.

## Supplementary Material

Supplementary figures.

Supplementary table S1.

Supplementary table S2.
