# Supplementary material for: Precise and efficient silencing of mutant KrasG12D by CRISPR-CasRx controls pancreatic cancer progression
Source: Theranostics. 2020 Sep 16;10(25):11507–19. doi: 10.7150/thno.46642 (PMC7545986; doi:10.7150/thno.46642)
Supplement: Supplementary file 1 — Supplementary figures. [file thnov10p11507s1.pdf]

Supplementary figure legends

Figure S1. (A) CasRx efficiently silences mCherry expression. 293T cells were transiently transfected by plasmids as indicated. Lipofectamine 3000 was used as described in methods. A fluorescence microscopy (Olympus) was used to capture the image. (B) Sanger-sequencing of Kras mRNA containing the region of G12D mutation. Total RNA was precipitated from indicated cells and reverse transcribed to obtain the cDNA as indicated in methods. Sequences were shown by SnapGene software.

Figure S2. (A-B) Histogram analysis of Kras intensity supplementary to Figure 2C. (C) CasRx overexpression did not affect the cellular Kras protein level. (D) A volcano plot of differentially expressed genes in PANC-1 cells ($Kras^{G12D}$ mutant cell) or (E) in H6c7 cells (Kras wild-type cell) transfected with CasRx+/gRNA+ targeting $Kras^{G12D}$. Genes with false discovery rate (FDR) <0.05 and |fold change| > 2 were set as differentially expressed genes (DEGs) and highlighted in red. Detailed list of DEGs from Figure S2D-E were shown in Table S1, S2.

Figure S3. CasRx-gRNA system did not affect MIAPaCa-2 cells bearing $Kras^{G12C}$ mutation. (A) RT-qPCR quantification of Kras mRNA. (B) Western-blot analysis of Kras protein. (C) Cell proliferation quantification by using CCK-8 assay. (D) Colony-forming assay in MIAPaCa-2 cells. (E) Gemcitabine IC50 determination in MiaPaCa2.

Figure S4. gRNA targeting mutant $Kras^{G12D}$ transcript silenced mutant $Kras^{G12D}$ transcript in AsPC-1 cells. (A) RT-qPCR quantification of Kras mRNA. (B) Western-blot analysis of Kras protein. (C) Cell proliferation quantification by CCK-8 assay. (D) Colony-forming assay in AsPC-1 cells. (E) Gemcitabine IC50 determination in AsPC-1. (F) RT-qPCR quantification of Kras mRNA in the orthotopic tumor as shown in Figure 4.

Figure S5. Supplementary to Figure 3G. (A) The morphology and Ki-67 staining of PANC-1 tumors. (B) The Mouse weight and (C) tumors weight. (D) CasRx and gRNA targeting mutant $Kras^{G12D}$ dramatically inhibited the 3D-proliferation of PANC-1.

Figure S6. Supplementary to Figure 5. IHC staining of p-Erk expression and the staining score statistics.

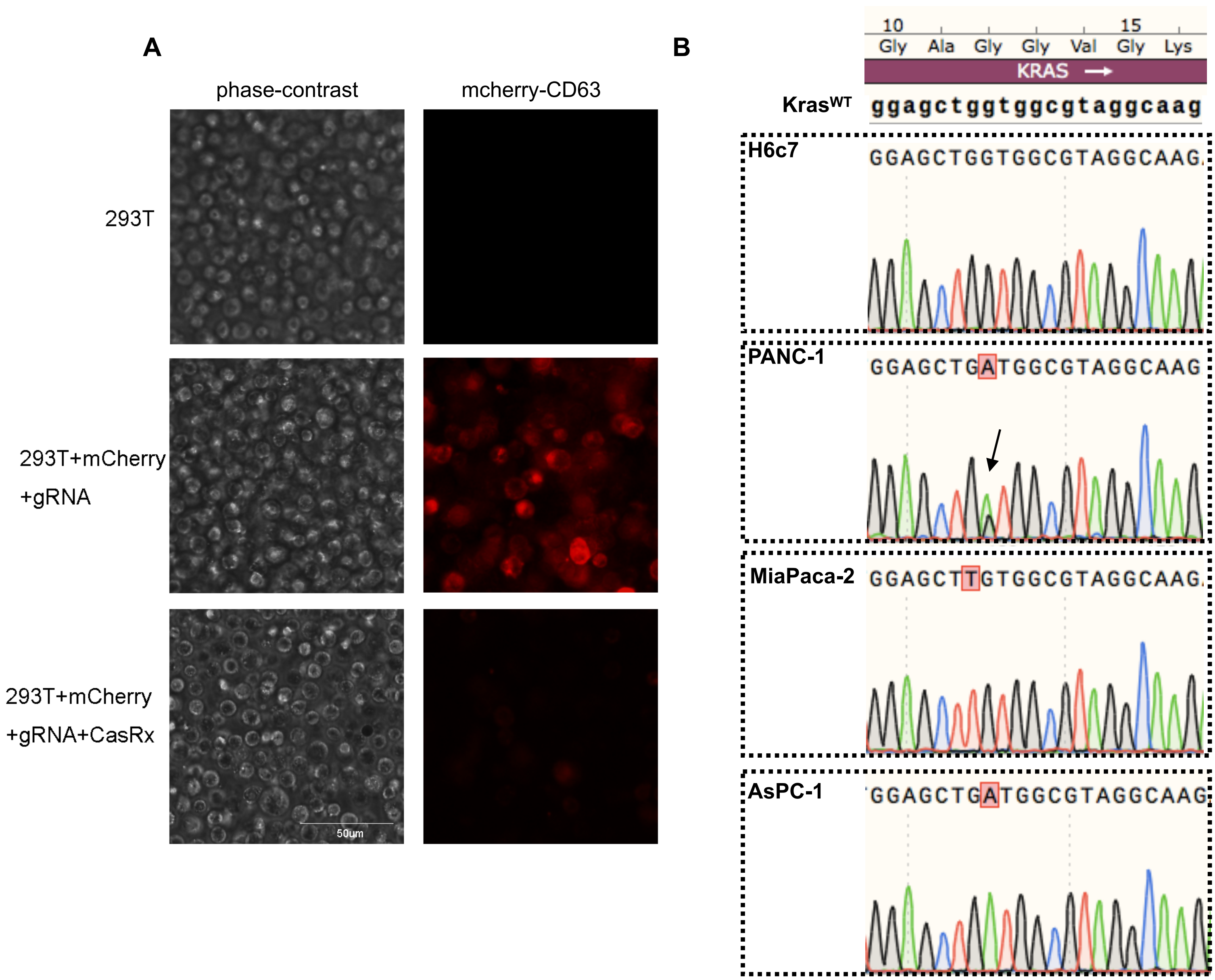
A
phase-contrast
mcherry-CD63
293T
293T+mCherry
+gRNA
293T+mCherry
+gRNA+CasRx
50um
B
10
15
Gly
Ala
Gly
Gly
Val
Gly
Lys
KRAS
Kras^WT
ggagctggtggcgtaggcaag
H6c7
GGAGCTGGTGGCGTAGGCAAG
PANC-1
GGAGCTGATGGCGTAGGCAAG
MiaPaca-2
GGAGCTTGTGGCGTAGGCAAG
AsPC-1
GGAGCTGATGGCGTAGGCAAG

Figure S1

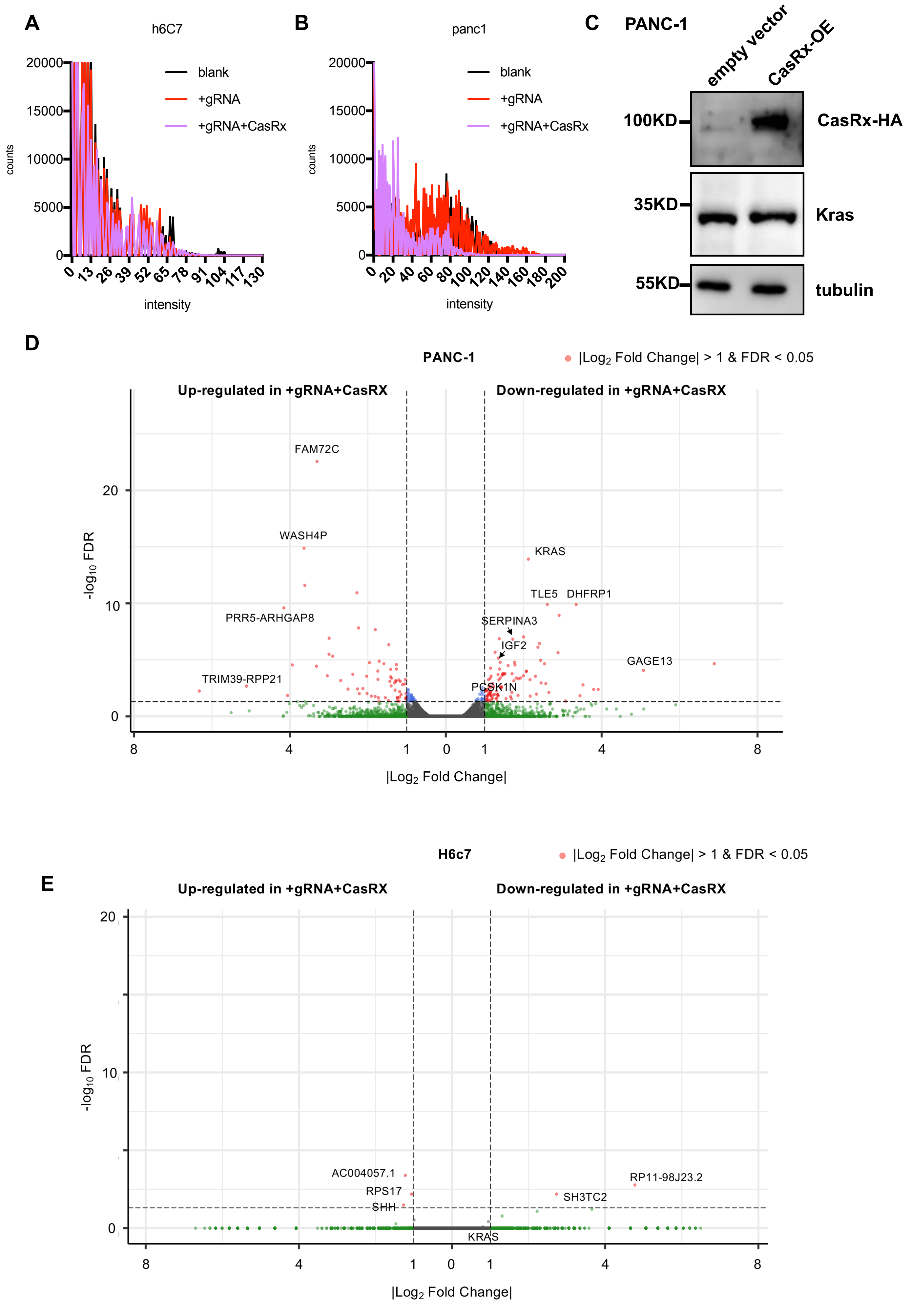
A
h6C7
blank
+gRNA
+gRNA+CasRx
counts
intensity
B
panc1
C
PANC-1
empty vector
CasRx-OE
100KD
CasRx-HA
35KD
Kras
55KD
tubulin
D
PANC-1
|Log2 Fold Change| > 1 & FDR < 0.05
Up-regulated in +gRNA+CasRX
Down-regulated in +gRNA+CasRX
FAM72C
WASH4P
PRR5-ARHGAP8
TRIM39-RPP21
KRAS
TLE5
DHFRP1
SERPINA3
IGF2
GAGE13
PCSK1N
-log10 FDR
|Log2 Fold Change|
E
H6c7
AC004057.1
RPS17
SHH
KRAS
RP11-98J23.2
SH3TC2

Figure S2

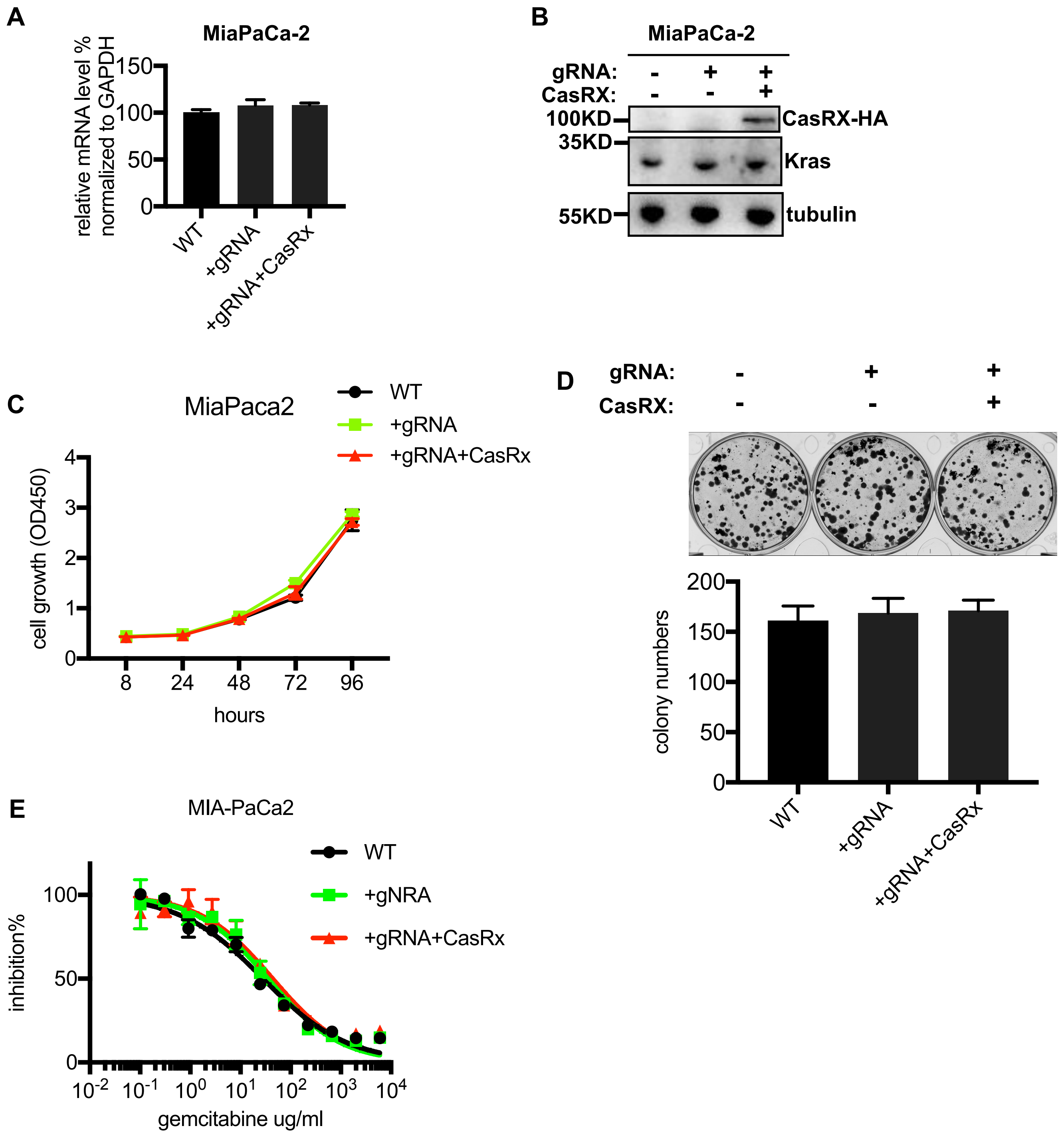
A
MiaPaCa-2
relative mRNA level %
normalized to GAPDH
150
100
50
0
WT
+gRNA
+gRNA+CasRx
B
MiaPaCa-2
gRNA: - + +
CasRX: - - +
100KD
CasRX-HA
35KD
Kras
55KD
tubulin
C
MiaPaca2
WT
+gRNA
+gRNA+CasRx
cell growth (OD450)
4
3
2
1
0
8
24
48
72
96
hours
D
gRNA: - + +
CasRX: - - +
200
150
100
50
0
colony numbers
WT
+gRNA
+gRNA+CasRx
E
MIA-PaCa2
WT
+gNRA
+gRNA+CasRx
inhibition%
100
50
0
10^{-2} 10^{-1} 10^{0} 10^{1} 10^{2} 10^{3} 10^{4}
gemcitabine ug/ml

Figure S3

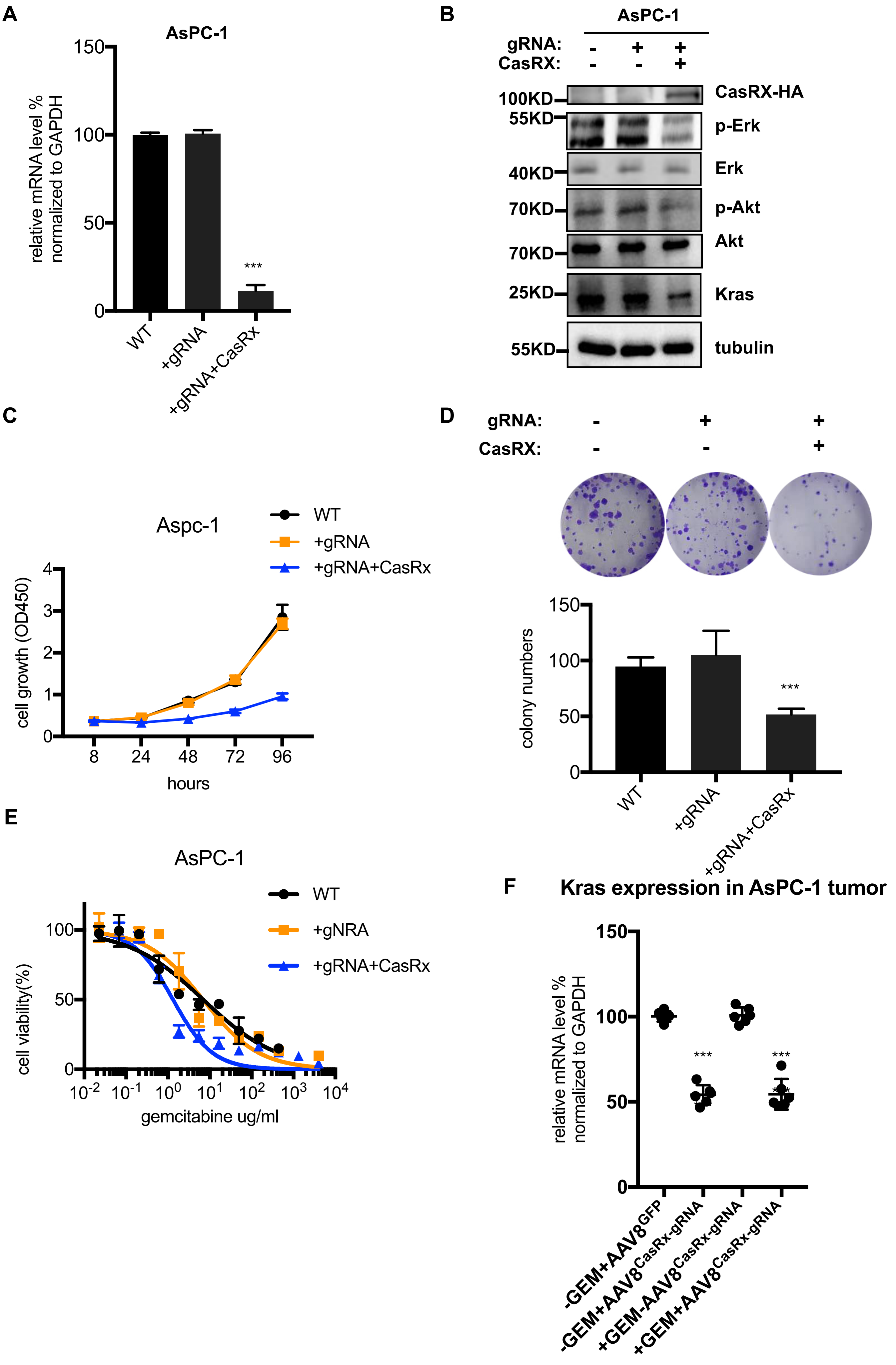
A
AsPC-1
relative mRNA level %
normalized to GAPDH
150
100
50
0
***
WT
+gRNA
+gRNA+CasRx
B
AsPC-1
gRNA: - + +
CasRX: - - +
100KD
55KD
40KD
70KD
70KD
25KD
55KD
CasRX-HA
p-Erk
Erk
p-Akt
Akt
Kras
tubulin
C
Aspc-1
WT
+gRNA
+gRNA+CasRx
cell growth (OD450)
4
3
2
1
0
8
24
48
72
96
hours
D
gRNA: - + +
CasRX: - - +
colony numbers
150
100
50
0
***
WT
+gRNA
+gRNA+CasRx
E
AsPC-1
WT
+gNRA
+gRNA+CasRx
cell viability(%)
100
50
0
10^{-2} 10^{-1} 10^{0} 10^{1} 10^{2} 10^{3} 10^{4}
gemcitabine ug/ml
F
Kras expression in AsPC-1 tumor
relative mRNA level %
normalized to GAPDH
150
100
50
0
***
***
-GEM+AAV8GFP
-GEM+AAV8CasRx-gRNA
+GEM-AAV8CasRx-gRNA
+GEM+AAV8CasRx-gRNA

Figure S4

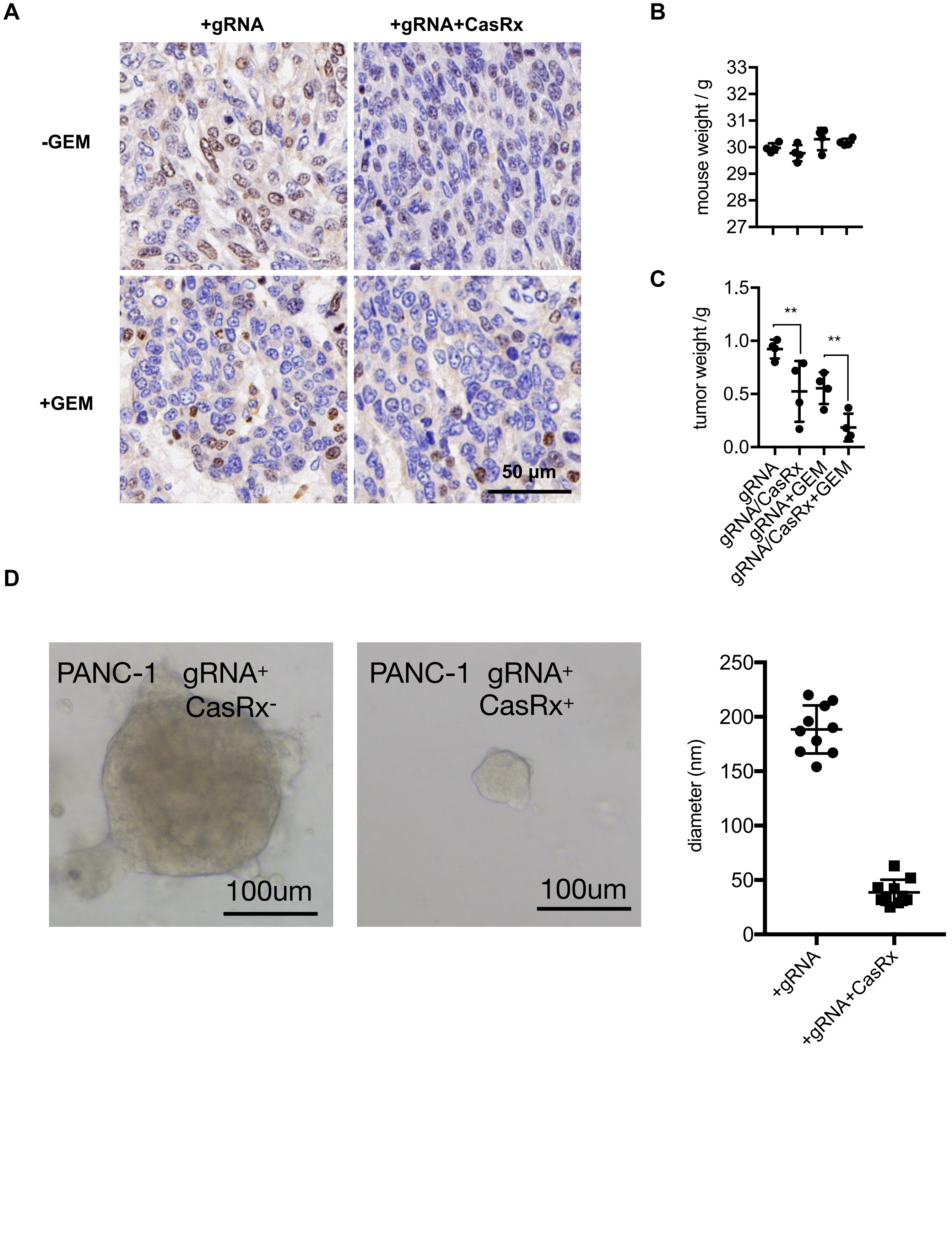
A
+gRNA
+gRNA+CasRx
-GEM
+GEM
50 μm
B
mouse weight / g
33
32
31
30
29
28
27
C
tumor weight /g
1.5
1.0
0.5
0.0
**
**
gRNA
gRNA/CasRx
gRNA+GEM
gRNA/CasRx+GEM
D
PANC-1 gRNA+
CasRx-
100um
PANC-1 gRNA+
CasRx+
100um
diameter (nm)
250
200
150
100
50
0
+gRNA
+gRNA+CasRx

Figure S5

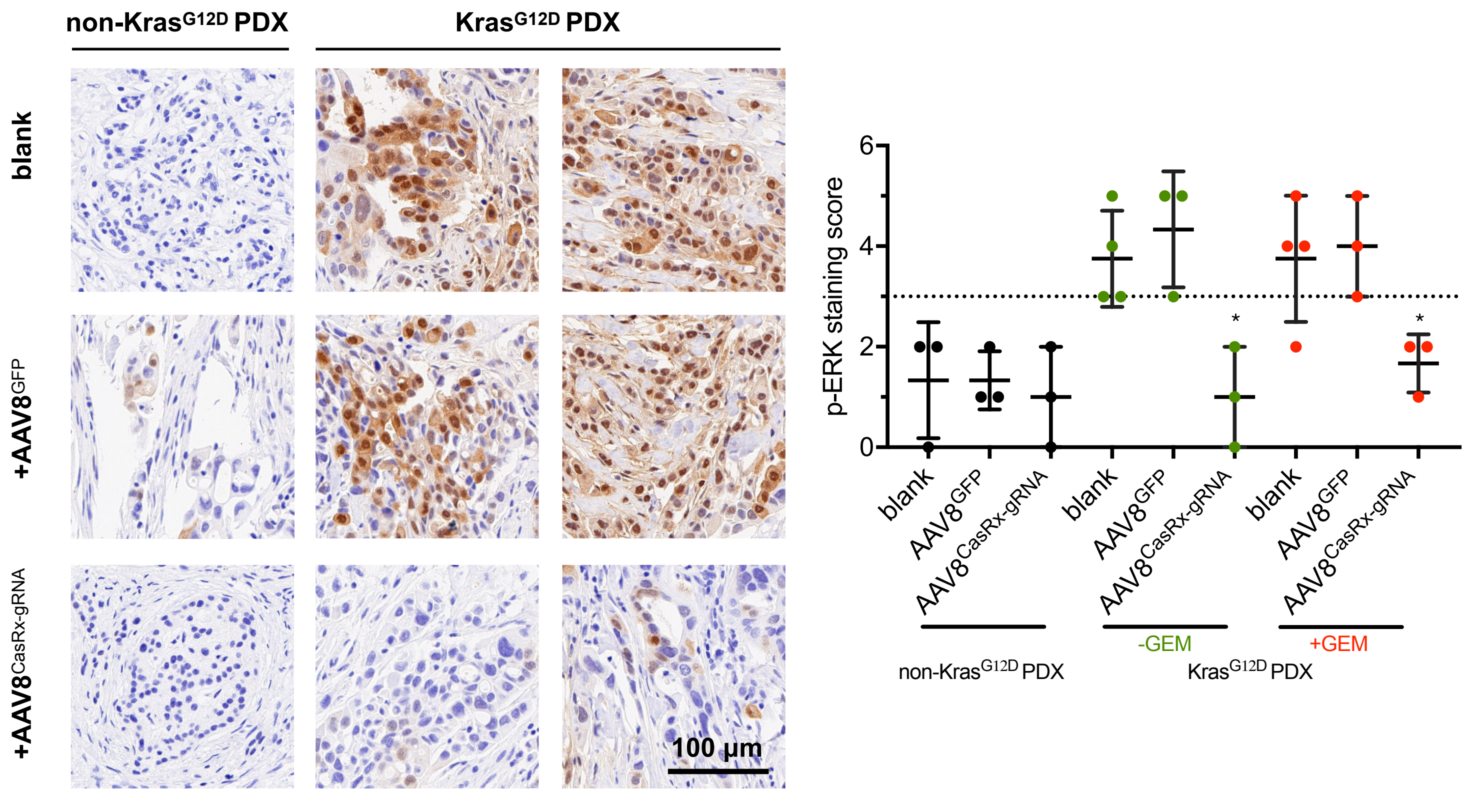
non-Kras^{G12D} PDX
Kras^{G12D} PDX
blank
+AAV8^{GFP}
+AAV8$^{CasRx-gRNA}$
100 μm
p-ERK staining score
6
4
2
0
blank
AAV8^{GFP}
AAV8$^{CasRx-gRNA}$
*
-GEM
+GEM
non-Kras^{G12D} PDX
Kras^{G12D} PDX

Figure S6
